# Exploring emergency physicians’ professional identities: a Q-method study

**DOI:** 10.1007/s10459-020-09973-y

**Published:** 2020-05-07

**Authors:** Yu-Che Chang, Xaviera Xiao, Nothando Nkambule, Roy Y. L. Ngerng, Alison Bullock, Lynn V. Monrouxe

**Affiliations:** 1 Chang Gung Medical Education Research Centre (CGMERC), Chang Gung Memorial Hospital, Linkou, Taiwan (R.O.C.); 2 Department of Emergency Medicine, Chang Gung Memorial Hospital, Linkou, Taiwan (R.O.C.); 3 Chang Gung University College of Medicine, Taoyuan, Taiwan (R.O.C.); 4 Risk Society and Policy Research Center, National Taiwan University, Taipei, Taiwan (R.O.C.); 5 Cardiff Unit for Research and Evaluation in Medical and Dental Education (CUREMeDE), School of Social Sciences, Cardiff University, Cardiff, Wales, UK; 6 The Faculty of Medicine and Health, School of Health Sciences, The University of Sydney, Sydney, NSW Australia

**Keywords:** Emergency physician, Emergency medicine, Professional identities, Q-methodology, Professional recognition

## Abstract

Professional identities research in medical education has made significant contributions to the field. However, what comprises professional identities is rarely interrogated. This research tackles this relatively understudied component of professional identities research by understanding emergency medicine physicians’ perspectives on the important elements that comprise their professional identities. Q-methodology was used to identify different clusters of viewpoints on professional identities; by extension, the core components that comprise emergency medicine physicians’ professional identities are disclosed. Thirty-three emergency medicine physicians were recruited, through purposive sampling, from five hospitals across Taiwan. R software was used to analyse the Q-sorts, determine loadings on each viewpoint and formulate the viewpoint array. Analysis of interview data enhanced our understanding of these viewpoints. In total, twenty-five emergency medicine physicians loaded onto four distinct viewpoints, reflecting dominant perspectives of emergency medicine physicians’ understanding of their professional identities. These distinct viewpoints demonstrated what emergency medicine physicians deemed significant in how they understood themselves. The viewpoints comprised: skills acquisition, capabilities and practical wisdom; coping ability and resilience; professional recognition and self-esteem; and wellbeing and quality of life. All viewpoints stressed the importance of trust between colleagues. These findings demonstrate the multitude of ways in which seemingly unified professional identities diverge across groups of individuals. An enhanced understanding of speciality work culture is gained. By understanding facets of professional identities, the development of future educational interventions and departmental initiatives, which might support key components of professional identities, can be explored.

## Introduction

Professional identities research in medical education rarely delves into what aspects—such as values, environment, artefacts or beliefs—of professional identities that physicians prioritize and consider salient. By contrast, investigations into the impact, the formation process and the measurement of professional identities tend to receive greater engagement (Allsop and Mulcahy 1998; Crossley and Vivekananda-Schmidt 2009; McNeil et al. 2013; de Lasson et al. 2016; Monrouxe 2016; Branch et al. 2017; Monrouxe et al. 2017). Notably, research has considered how professional identities may influence a wide range of attitudes on issues pertaining to ethical standards, new technology adoption in the clinical context (Mishra et al. 2012; Keller et al. 2016) and professional commitment (Molleman and Rink 2015; van Os 2015). In terms of developmental issues, professional identities formation research explores how medical students and junior doctors’ professional identities are impacted by early formative experiences and can be developed through educational initiatives aimed at fostering professional identities (de Lasson et al. 2016; Monrouxe 2016; Monrouxe et al. 2017; Kalet et al. 2018). While the contributions of this body of work should not be overlooked, understanding the core question of what encompasses physicians’ professional identities is relatively under investigated.

Drawing on social constructionism (Rees et al. 2019), identities theorists contend that identities comprise a mutable process: they are informed by a variety of values and beliefs derived from one’s environment, mediated through language and artefacts, and influenced by the many other identities that an individual holds (Jenkins 2008), which in turn are also imbued with values and beliefs. Identities are also interactional. The specific context or unique interpersonal dynamics at play might impact the expression of one’s identities (Rees and Monrouxe 2018). The accumulation of one’s experiences matter, as identities may evolve based on a variety of values and beliefs (Jarvis-Selinger et al. 2012). In effect, “identities give our lives meaning” (Rees and Monrouxe 2018).

In the case of medical education, Monrouxe draws on sociological and anthropological theories as she elaborates “(d)eveloping an identity as a doctor is achieved through the process of identification: a two-way, internal and external process whereby we define ourselves (who I think I am) and are simultaneously defined by others (who I think you think I am)…” (Monrouxe 2009). As this definition underscores, professional identities encompass complex and diverse components such as personal histories, core values and aspects of one’s work culture. By that same token, artefacts such as attire, organizational relationships and employment circumstances may also be components that frame how one thinks about professional identities. These facets that may shape physicians’ professional identities are illustrative, not exhaustive.

Drawing on the notion that professional identities comprise various components, such as values, beliefs and environment, our research seeks to explore what is emphasized when emergency medicine physicians—a contingent of specialist physicians who have been underrepresented in professional identities research—are asked to describe their professional identities. In doing so, our research asks for following question:

RQ: What are emergency medicine physicians’ perspectives on what aspects comprise their professional identities?

From a theoretical standpoint this bears significance in medical education insofar as it aims to interrogate physicians’ professional identities, a critical concept that is used widely within the discipline. Moreover, understanding the dominant perspectives surrounding professional identities of a contingent group of physicians, who face daily occupational challenges (Heng 2014) and report high levels of stress (Arora et al. 2013), can be generative. Such an inquiry might provide insight on what professional identities tell us about how challenges, rewards or simply the daily demands of a medical speciality might be interpreted. And, in turn, can provide insights into professional identities with which individuals from other medical specialties might also identify.

Our research sets forth to explore the dominant beliefs that emergency medicine physicians hold regarding their professional identities by utilizing Q-methodology. While Q-methodology’s specificities will be explained in greater depth below– it is significant to note that Q-methodology’s utility lies in its capacity to engage in the richness qualitative research offers, while also relying on quantitative tools, to find a systematized way to develop a taxonomy of viewpoints on (in this case) emergency medicine physicians’ professional identities.

## Methods

### Study context

The study is part of a larger multi-year mixed methods study of burnout, resilience and the professional identities of emergency medicine physicians in Taiwan. Taiwan’s National Health Insurance (NHI) provides heavily subsidised services. As a result, visits to the Emergency Department are relatively low-cost and, consequently, oversubscribed. The hospitals within this study are part of an extensive private hospital network that serves one tenth of Taiwan’s health care needs. It is noteworthy that this study was executed towards the culmination of a collective resignation incident, wherein over 30 emergency medicine physicians left their positions, although some ultimately returned. Although the timing of our data collection was not designed to coincide with this particular incident, we are mindful that their identities—the very values people held as emergency physicians—were at the fore during this time.

### Study design

Q-methodology combines quantitative and qualitative findings to understand subjectivity (Watts and Stenner 2005). Q-methodology encompasses Q-sorting—a card sorting exercise—and posting-sorting interviews. Participants are given set of statement cards (a Q-set), which are developed specifically for the study, to place individually on a (typically) normal distribution-shaped grid according to their preference. In our study participants were asked to rank the Q-set statements based on the statements’ relevance (or lack thereof) to the following question: thinking about your experience as an emergency physician, what values and beliefs shape how you feel about yourself as an emergency physician?

While Q-methodology research does not require the use of a forced choice normal distribution grid, research suggests it makes almost no difference in the results (Brown 1980). Given that many Q-methodology practitioners argue that its inclusion does simplify the process for participants, we opted to use this method (rather than free distribution). All materials, which included Q-sorting cards and interview guides, were prepared in English and Chinese. Participants’ interviews were conducted in Chinese. The research team comprised a Taiwan emergency physician with clinical teaching and research experience, a statistician, medical education scholars and research assistants. The majority of the research team are proficient in both English and Mandarin Chinese.

#### Q-set development

The development of Q-set statements was undertaken in accordance with Q-methodology recommended practice. Firstly, the Q-set statements were drawn from *the concourse* comprising “all the manifestations and expressions of human response and dialogue, verbal and nonverbal” (Wilson 2005) that relate to the topic under study. Given this, the concourse can be derived from a wide variety of materials including relevant literature, popular media, interviews, informal conversations, and purposive interviews (Ward 2010). Secondly, the Q-set statements’ value and utility lie in their ability capture the diverse range of ideas that comprise the concourse. As such, a classification system can facilitate this objective because it provides a systematic way for the research team to ensure that distinct and disparate elements of the concourse are represented in the Q-set (Stenner 2012).

In our study, we conducted a literature review of physicians’ (general) professional identities alongside one-to-one interviews with n = 25 emergency medicine physicians (specific) on the subject of their own professional identities to serve as the content material for our concourse. Drawing on interviews and literature reviews to develop the Q-set is a common practice within Q-methodology studies (Watts and Stenner 2005; Ward 2010). Following this, our classification system comprised a thematic framework analysis that was subsequently applied to both literature review and interview data (Richie and Spencer 1994). The research team collectively identified themes according to this 5-step method that included individual team members undertaking a close reading of a subset of data before coming together to identify and agree on themes/sub-themes. All data were managed using EndNote X9 (for articles) and ATLAS.ti (for article/transcript coding) software. Ten themes were identified: personal traits; professional development and medical training; work style and management; physician–patient relationship; coping skills; emergency medicine as a unique specialty; research and teaching; team building and leadership; hospital support; and external support. After a series of further discussions amongst the research members (who comprise social science researchers and emergency medicine physicians), 54 statements, derived from the 10 themes, were chosen (Table 3). We did not pilot these items to determine their utility. Rather, the utility of the Q-set came from the statements reflecting diverse components of the topic, as derived from: (a) research that focused on physicians’ identities generally; (b) interviews with emergency medicine physicians around their identities specifically; and (c) a diverse research team classifying both data sets via regular discussions and proactive engagement.

### Participant sample

Thirty-three emergency medicine physicians (seven resident physicians and twenty-six attending physicians) participated. Emergency medicine physicians responded to a notice circulated across the Emergency Departments of five hospitals. Emergency medicine physicians who joined the previous study underpinning the Q-sort development (described above) were ineligible. Purposive sampling was deployed in order to ensure there was adequate inclusion of female emergency medicine physicians and those across different career stages.

Ethical approval was obtained by the participating hospital group. Participation was voluntary, informed written consent was gained and respondents’ anonymity is protected. Power relations between researcher and participants were not of concern as the research assistant conducting the exercises did not have an intimate or even professional engagement with the emergency department as an entity. The study received national funding. Respondents were compensated for their time.

### Procedure

#### Q-sort and interviews

**Fig. 1:**
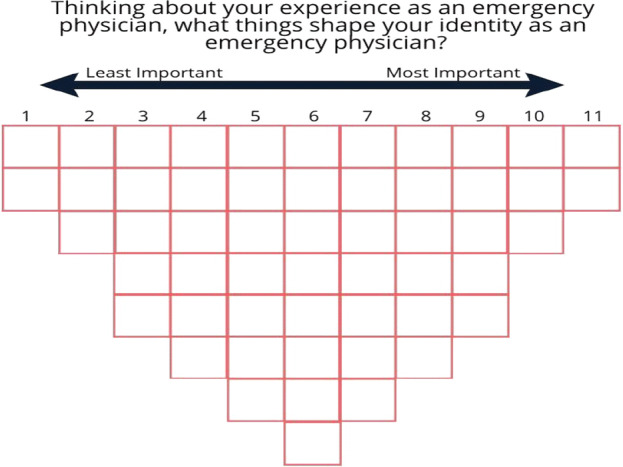
The forced choice distribution grid

The one-to-one Q-sort exercises and post-sorting interviews were led by a research assistant (RN) and completed between July - September 2017. Respondents were asked to think about their experience as an emergency physician, and what shapes their professional identities as an emergency physician. A definition for professional identities was only provided when participants inquired. The researcher relayed that the research team defined professional identities as “who you are and who are seen to be by others”. Respondents answered the question by placing the Q-set statements cards, on a forced choice distribution grid with 54 empty slots (Fig. 1). Before placement, respondents differentiated the cards based on three categories: important, not important and neutral. The grid ran on a continuum of “least important” (1) from the left to “most important” (11) on the right.

Immediately after the Q-sorting exercise, post-sorting semi-structured interviews were conducted. Respondents answered a series of questions about the sorting process, which included explaining why statements were considered “most important” and “least important”. The interviews were recorded, transcribed, anonymised and linked to their unique Q-set.

### Data analysis

The thirty-three Q-sorts were analysed using R software. Unlike quantitative research methods, in the Q-methodology, the research respondents are the *variables*. As such the correlation matrix reflects the connection between different Q-sort patterns (participants’ perspectives). R deploys principal component analysis to determine factor loadings: for the sake of clarity, the term *viewpoint* will mostly be used in substitution of the term *factor*. In terms of selecting a rotation method, Q-methodology practitioners explicitly argue the importance of choosing a rotation method that yields the clearest structure for interpretation (Zabala and Pascual 2016). Varimax rotation was used (rather than quatimax, oblimin, or promax) as it generated the clearest viewpoint extraction for interpretation (Ramlo 2016; Akhtar-Danesh 2017) and yielded more distinguishing statements with which to work. As such, the use of varimax rotation was able to improve our understanding of the different viewpoints on professional identities. To qualify as a viewpoint, the eigenvalue must be above 1.0 and each viewpoint needs to include more than three Q-sorts. In order to determine how many viewpoints are most appropriate, a degree of human judgment is necessary (Damio 2018). Essentially, the viewpoints comprise a cluster of Q-sorts based on the similarities of the patterning of statement ranking. Thus, it is important to remember that viewpoints reflect how participants *think* about their professional identities in terms of what they value, rather than them being about traits, physical aspects, or psychological assessments.

After determining the appropriate number of viewpoints, see below, the viewpoint array was analysed. The viewpoint array determines the weighted average of the Q-sorts from the viewpoints and indicates where each statement from the Q-set is placed on the grid. The viewpoint array does not merely indicate what statements are ranked “most important” and “least important”; it indicates each viewpoints’ distinguishing statements: those statements that are uniquely important and reflective of a single viewpoint. The interview analysis aimed to illuminate the distinguishing statements and better understand important and least important statements in order to reveal the perspectives expressed by each viewpoint.

## Results

Upon analysis and discussion, the research team determined that four viewpoints appropriately represented prominent and distinct perspectives on professional identities. This included 25 of the 33 (76%) Q-sorts. Each eigenvalue value was between 4–5 and the total variance was 54.08% (Table 1), which is considered appropriate (Stenner 2012).

**Table 1:** Eigenvalue and variance for the 4 factors

Factor (# of respondents)	Eigenvalue	Variance explained (%)
1 (7)	4.91	14.87
2 (5)	4.55	13.78
3 (6)	4.34	13.16
4 (7)	4.05	12.26

Each viewpoint was named based on the values and ideas that were prioritised in respondents’ explanations of professional identities: Viewpoint 1 skill acquisition, capabilities and practical wisdom; Viewpoint 2 coping ability and resilience; Viewpoint 3 professional recognition and self-esteem; and Viewpoint 4 wellbeing and quality of life. The demographic details of each viewpoint are outlined (Table 2) and the factor array (Table 3) demonstrates the placement of each Q-set statement, in terms of where they would be placed on the distribution grid, for each viewpoint. Following this, each viewpoint is explained and excerpts from the interview data are provided where appropriate.

**Table 2:** Demographic detail of the four viewpoints

	Total	Viewpoint 1	Viewpoint 2	Viewpoint 3	Viewpoint 4
# of respondents	25	7 (28%)	5 (20%)	6 (24%)	7 (28%)
Age					
Range	29–50	29–50	31–39	40–49	30–47
Average	38	38.4	34.8	43.5	37
Years in EM (avg.)	11.96	11.1	8.4	17.5	10.6
Sex					
Male	19 (76%)	5 (71.4%)	3 (60%)	6 (100%)	5 (71.4%)
Female	6 (24%)	2 (28.6%)	2 (40%)	0	2 (28.6%)
Non-clinical work					
Teaching	19 (76%)	5 (71.4%)	3 (60%)	6 (100%)	5 (71.4%)
Research	13 (52%)	5 (71.4%)	2 (40%)	3 (50%)	3 (42.9%)
Married	21 (84%)	5 (71.4%)	5 (100%)	5 (83.3%)	6 (85.7%)
Rank					
Attending	19 (76%)	6 (85.7%)	3 (60%)	6 (100%)	4 (57.1%)
Resident	6 (24%)	1 (14.3%)	2 (40%)	0	3 (42.9%)

**Table 3:** Factor arrays of the four viewpoints

#	Statements	Viewpoint 1	Viewpoint 2	Viewpoint 3	Viewpoint 4
1	My personal values (what I consider as valuable and important in my life)	5	10	8	10
2	Being given tasks that are relevant to the work of an emergency physician	6	4	5	5
3	Working shifts	2	3	2	4
4	Being able to handle patients who are violent towards me	6	8^a^	4	4
5	Being able to expand my skills in a sub-specialty within emergency medicine	6	5	9^a^	3
6	Having a good department director	2	9^a^	6	4
7	Taiwan’s National Health Insurance scheme	1	4	1	2
8	Feeling happy with my personal life	5	6	7	11^a^
9	Wearing the emergency physician attire	1	2	3	3
10	Having a challenging patient	8^a^	2	3	3
11	Experiencing the death of a patient for the first time	3	1	4	1
12	When the public recognises emergency medicine is a specialty in its own right	7	6	11^a^	4
13	Having the autonomy to make decisions at work	7	9	11	5
14	The important role that emergency physicians play in society	6	1	9	9
15	Having an extroverted personality	4	3	2	6
16	Having knowledge of emergency medicine	8	7	5	7
17	Being able to communicate well with my patients	7	6	6	8
18	Receiving feedback from patients about my work	4	4	4	6
19	Experiencing the fresh challenges that emergency medicine brings everyday	10^a^	5	6	7
20	Being in a senior emergency physician role	5	4	5	2
21	Being able to find a balance between my work and personal/family life	4	11	9	11
22	Having high emotional intelligence (EQ)	6	8	8	9
23	Becoming experienced in my work as an emergency physician	8	9	6	5
24	Seeing patients get well under my care	6	4	6	8^a^
25	Taking time out to reflect on events at work	7	5	5	5
26	Doing research	3	4	3	1
27	When the hospital recognises the contributions of emergency physicians	6	6	8^a^	5
28	Being able to enjoy my work as an emergency physician	7	9	10	8
29	Being physically strong	3	7^a^	3	5
30	Feeling competent as an emergency physician	8	7	10^a^	7
31	Being able to work under pressure	9	11^a^	7	7
32	When a role model says that I am a good emergency physician	5	3	6	6
33	Taking on a teaching role	5	3	9^a^	3
34	Having adequate staffing in the emergency room (ER)	4	8	7	5
35	Having personal hobbies and engaging in leisure activities outside of work	4	5	6	10^a^
36	Going through the long residency training	3	3	3	4
37	Being able to multitask	10^a^	7	8	6
38	Not making clinical errors	9	8	1	6
39	Being able to diagnose a medical condition quickly	11^a^	7	6	8
40	Working in a team with other emergency physicians	9	6	8	9
41	Working at a medical centre	3	2	2	2
42	When my salary increases	2	8	5	7
43	Passing the board examination for emergency medicine	4	5	4	6
44	Being able to solve problems quickly	10	7	7	10
45	Being able to manage sudden events that arise at work	7	10^a^	7	7
46	Being able to find other ways of managing illnesses which physicians from other specialties cannot	11	6	10	4
47	Being able to trust the colleagues that I work with	9	9	7	8
48	When a professional association representing emergency physicians speaks up for their rights	6	6	8^a^	3
49	Learning to act and behave like an emergency physician from my emergency physician co-workers	8^a^	5	4	6
50	Having a manageable workload	7	8	4	9
51	Being able to recover quickly from things that upset me at work	5	7	5	6
52	Being able to provide holistic and comprehensive care for my patients	8	5	7	8
53	Being a good team leader in the clinical setting	5	6	9	9
54	Being able to optimise patient flow in a timely manner when the emergency room is overcrowded	9	10	5	7

^a^Distinguishing statements

### Viewpoint 1: Skill acquisition, capabilities and practical wisdom

Skill acquisition, capabilities and practical wisdom comprised six attending physicians and one resident physician: 28% of the respondents. They ranged from ages 29–50. Distinguishing statements comprise *being able to diagnose a medical condition quickly*, *experiencing the fresh challenges that emergency medicine brings every day*, *being able to multitask*, *having a challenging patient* and *learning to act and behave like an emergency physician from my emergency physician co*-*workers*.

During interviews with those whose Q-sort was grouped in this viewpoint, participants highlighted how possessing the skills necessary to tackle the challenges and unpredictable environment of the ED underpins their professional identities:

EM brings new challenges every day. That’s actually why I choose EM in the first place, I really wanted to see a variety of patients, and patients who weren’t necessarily easy to figure out. Whenever a patient comes to me, he might suffer from chest pain, abdominal pain, or have difficulty breathing…(I)t’s up to me instead of others to find the answer. (34, Female, Attending Physician)

Beyond meeting the demands of the ED, participants from this viewpoint also stressed the difference in expertise between emergency medicine physicians and non-EM trained physicians:

We (EM Physicians) are able to integrate (knowledge from) all medical specialties…(s)o the way we make diagnoses is certainly different from other physicians. Because of this we are typically able, under a short period of time, to see patients and figure out what their problem is and what needs to be done. (29, Male, resident physician)

Notably *being able to find other ways of managing illnesses which physicians from other specialties cannot* also ranked high. Being able to practice their distinctive skills was described as a source of pride, as it reflects the practical wisdom of an EP. The distinguishing statement *learning to act and behave like an emergency physician from my emergency physician co*-*workers* further affirms the importance placed on the skills and work culture that differentiates emergency medicine physicians.

It is significant that emergency medicine physicians from this viewpoint tend to deemphasise elements outside of clinical practice. For instance, *(m)y personal values (what I consider as valuable and important in my life)* (ranked:5) and *being able to find a balance between my work and personal/family life* (ranked:4) are both statements that highlight personal identities and commitments that ranked especially low, particularly in comparison to the other viewpoints. This also extends to how these emergency medicine physicians consider that the public and patients’ perceptions of their discipline are separate from how they understood their professional identities:

…(T)he health insurance system, the training method, research, personnel changes, and (board certified) tests you take, I am of the belief that no matter how much these things change, the way you approach the profession will remain the same. It’s not as if a good director isn’t important, rather if you’re looking at professional identities, I think all these other factors, I really don’t think they have a strong impact. (29, Male, resident physician)

The lack of attention placed on these emergency medicine physicians’ non-professional identities and commitments, the health care system and external perceptions of their profession reinforces that they tend to understand their professional identities by way of their performance and capabilities.

### Viewpoint 2: Coping ability and resilience

Coping ability and resilience comprised three attending physicians and two resident physicians, 20% of the respondents. These participants occupied a specific age range: 30–39. Distinguishing statements comprise *being able to work under pressure*, *being able to manage sudden events that arise at work*, *having a good department director*, *being able to handle patients who are violent towards me* and *being physically strong.*

Participants’ descriptions of the emergency department fleshed out the imagery of stress connoted in the distinguishing statements:

Because EM Physicians’ work is so stressful, so I feel being able to adapt to a high stress work environment is a priority…because every day is stressful…and the source of the stress is the sudden events…you have to be able to handle the situations, including communicating with the patients and their family members. Sometimes, the patients will just suddenly die, which is the most stressful, the biggest thing that can happen. Then just every day and at any time, any kind of patient can come in. No one really knows. (32, Male, resident physician)

When describing the taxing elements of EM, many emergency medicine physicians from this viewpoint emphasised the importance of physical strength and being able to take on night shifts, which was associated with being in the early stages of their careers and being relatively young:

…even though work is exhausting, I still have enough energy to follow up on patients or to see if the patients’ illnesses are caused by other factors. I think this is such an important element of my development in emergency medicine… if every time I came to work and finished and just went right to sleep and didn’t really think about if there was more things I could do, honestly speaking I think in terms of my development… if I have time to really think and research (what I’ve seen) it’s going to help my growth as a physician. (31, Male, resident physician)

Beyond emphasising the ability to perform under pressure, having strong department leadership was considered especially important amongst these physicians. The significance of *having a good department director* lay in the belief that a positive relationship with hospital administration would provide them with a sense of career direction and adequate support, particularly during periods of uncertainty, as highlighted in our study context section:

Originally we also had some rumours spreading, but our director was able to calm everyone and was able to negotiate certain policies with the hospital. That’s when everything came to an end and that’s also the reason why we didn’t make more complaints. (31, Male, resident physician)

Finally, in line with Viewpoint 1 (skills), these physicians emphasised the clinical environment. *The important role emergency medicine physicians play in society*—which ranked high amongst participants from other viewpoints—was considered one of the least important statements (ranked 1) for participants within Viewpoint 2 (coping):

…I am not sure what kind of social role that I play, I don’t really know. As an EM Physician, I don’t really think I have the responsibility to make a contribution to society… I feel like what’s really necessary is to be in the hospital properly taking care of patients and that’s it. (34, Female, attending physician)

### Viewpoint 3: Professional recognition and self-esteem

Professional recognition and self-esteem comprised six (28%) attending physicians, 24% of the respondents. In contrast to Viewpoint 2 (coping) participants, this group of physicians were between 40 and 49 and occupied a comparatively late career stage. It is also notable that all participants from this viewpoint are male. However, due to the lack of female emergency medicine physicians over 40 in our sample, our study cannot make any remarks about why this viewpoint lacks gender diversity. Distinguishing statements comprise *when the public recognises emergency medicine is a specialty in its own*, *feeling competent as an emergency physician*, *being able to expand my skills in a subspecialty within emergency medicine*, *taking on a teaching role, when the hospital recognises the contributions of EP* and *when a professional association representing emergency medicine physicians speaks up for their rights*.

As the distinguishing statements imply, these participants found value in having their work as emergency medicine physicians recognised within the hospital and by the larger public, insofar as better recognition of emergency medicine was considered a factor that could improve how hospital administration might treat the emergency department.

Society’s general perception (of EM) will impact the hospital’s decision making (40, Male, attending physician)

In line with this logic, poor recognition of emergency medicine was considered to have a negative impact on the professional partnerships between the emergency department and other departments in the hospital:

In fact, (we) can’t just work with EM staff. We also need to collaborate with other specialties…so if other specialties recognise our specialty, it would be useful for us to work together from an operational perspective. (45, Male, attending physician)

These participants also believed that widespread poor understanding of emergency medicine contributed to oversubscription of their services:

I believe that the public needs to be educated, so if they were educated they would understand what kind of profession emergency medicine is and how it’s different than just a general clinic…then (the public) wouldn’t just come to the EM because of small issues. (44, Male, attending physician)

Beyond improving partnerships with other departments and the emergency department’s status within the context of the hospital, better recognition of emergency medicine was also important given the role emergency medicine physicians play within a larger societal context:

I see myself as an EP that society needs. It’s not about how I see myself, but about what the society needs me to be. (43, Male, attending physician)

Gaining recognition and establishing their role within society positively impacted how participants from this viewpoint felt about themselves:

… (I)t doesn’t matter who you are professionally, if others are able to recognize you as a professional, people will respect you (44, Male, attending physician)

The importance of emergency medicine recognition is not surprising when taking into account that these participants frequently discussed how their outward facing non-clinical work, which included teaching and developing partnerships with outside health bureaus, the fire department and other emergency service providers, enhanced their sense of professional identities:

EM does something that other departments just can’t do. So if the public is outside the hospital, it’s (EM) like a bridge that connects them with other medical specialties in the hospital. And when it comes to things that happen outside the hospital such as pre-hospital care and emergency medical service (EMS), only EM physicians can do that. We also work as EMS instructors, we have good relationships with the fire department and the health department…other departments just can’t do this, so this is really what distinguishes EM from other specialties. (40, Male, attending physician)

Similar to emergency medicine physicians from Viewpoint 1 (skills), *being able to find other ways of managing illnesses which physicians from other specialties cannot* was an important statement for Viewpoint 3 (recognition) participants, however for notably disparate reasons. Viewpoint 3 (recognition) participants place a premium on recognition and the important societal role of emergency medicine; participating in the clinical environment is merely one component of emergency medicine. This stands in contrast to the former groups who place a strong emphasis on the specific environment of the emergency department when describing their professional identities.

Finally, *not making clinical errors* is listed as a least important statement in Viewpoint 3 (recognition: ranked 1), which is noteworthy given its relative importance in Viewpoint 1 (skills: ranked 9), Viewpoint 2 (coping: ranked 8) and Viewpoint 4 (wellbeing: ranked 6). When taking into account that all emergency medicine physicians from Viewpoint 3 (recognition) have teaching experience and their relatively extensive careers in emergency medicine, their comparative tolerance for mistakes is understandable. Namely, mistakes were recognised as an inevitable consequence of having a long career in medicine, not a marker of incompetence; moreover, mistakes could be interpreted as teachable moments to share with less experienced emergency medicine physicians:

I draw on the mistakes I’ve made when speaking with younger doctors and tell them not to make the same mistakes that I’ve made. I discuss why I made these mistakes, how I misunderstood the patient, how I misread the symptoms, what I failed to do at the time that led to mistakes. I hope that they won’t encounter the same problems next time… Likewise, I can also learn from others as well. (44, Male, attending physician)

In effect, these physicians place value in understanding their role within a larger context, which is evidenced by their contributions as teachers, by developing professional partnerships outside of the hospital and by the explicit argument put forth about the significance of recognition for emergency medicine. While Viewpoint 2 (coping) participants highlight the importance of strong leadership and contemplate career trajectories, Viewpoint 3 (recognition) participants’ professional identities reflect the priorities of physicians at a later career stage. These physicians have been given the opportunity to explore EM outside of the clinical environment and, subsequently, interpret their professional identities in a manner that reflects its place within a larger societal and health care context.

### Viewpoint 4: Wellbeing and quality of life

The wellbeing and quality of life viewpoint comprised four attending physicians and three resident physicians. They comprised 28% of the respondents and encompassed a wide age range: 30–48. Distinguishing statements that highlight work-life balance comprised: *having personal hobbies* and *engaging in leisure activities outside of work* and *feeling happy with my personal life*. *Seeing patients get well under my care* was the only distinguishing statement that addresses work commitments.

For these emergency medicine physicians non-work commitments such as family and hobbies were prioritised:

I think personally, you’re not your job. So if I’m looking at my day, 24 h, I feel that striking a balance between personal time and family is very important. Furthermore, I want my life to be happy which, in a way, motivates me to keep living… (s)o I think it’s the most important. (31, Female, resident physician)

Participants from this viewpoint also elaborated that they choose emergency medicine because, unlike other departments, physicians are not put on call, which ensures that they can completely relax during their time-off. Having this clear distinction between work and life was considered an important factor that contributed to their success at work:

When you’re working you’re completely wholehearted, you’re only focused on work. So then when you’re off work, of course sometimes you think about work, but compared to doctors from other specialties, it’s really quite different. You have more clear-cut time to really engage in your own life and make plans. When there is a real opportunity to relax, recharge, when you’re back at work you’re less likely to experience fatigue. (30, Male, resident physician)

Maintaining this balance also helped mitigate work stressors:

… if the patient gets out of hand and so do his/her family members, it’s going to impact your clinical judgment because in your subconscious you won’t want to be around this person and won’t want to have any contact with this person. Even if I’m supposed to touch his belly, I won’t be willing to do so. I feel that the basic principle for me is that though I can’t control others, I can control myself. Due to this, I will maintain physical and mental peace so that I can have empathy for that kind of patient, patiently communicate with them, and develop a good doctor patient relationship. Therefore, I can more objectively judge my patient’s situation and give this patient the best decision making. (31, Female, resident physician)

Finally, emergency medicine physicians within Viewpoint 4 (wellbeing) are also notable for emphasising positive interactions with their patients, which is reflected in the distinguishing statement *seeing patients get well under my care*:

…of course the ultimate goal is that your patient’s condition improves, which is also our most important goal…therefore physicians societal role and improving the patient’s condition are the most important. (31, Male, resident physician)

Being able to improve patients’ conditions and prioritise their own self-care are integral components of how these emergency medicine physicians understand their professional identities.

### Least important statements

*Wearing the emergency physician attire*, *experiencing the death of a patient for the first time*, *doing research* and *working at a medical centre* were considered unimportant amongst all the viewpoints*. Taiwan’s National Health Insurance (NHI)* was also given a low ranking, despite being discussed by emergency medicine physicians from all viewpoints. NHI was deemed a source of frustration; many emergency medicine physicians attributed the regular oversubscription of emergency medicine with the low cost of healthcare services made possible through NHI. Nevertheless, NHI was deemed an unavoidable element of being a physician in Taiwan; it was beyond their ability to change. This suggests that although emergency medicine physicians claimed that NHI is an insignificant element of their professional identities, this does not mean that they do not care about the NHI or feel impacted by its effects.

### Consensus statements

Finally, *being able to trust the colleagues that I work with* was the sole consensus statement. The significance of trust was emphasised consistently and often associated with maintaining a strong sense of teamwork. The shift nature of the emergency department requires that emergency medicine physicians hand off patients and tasks to their colleagues; in order to maintain a smooth operation, it is necessary to feel confident that fellow staff are competent.

## Discussion

Our Q-methodology study contributes to a stronger understanding of how to study physicians’ professional identities by exploring emergency medicine physicians’ professional identities, wherein we uncovered four dominant viewpoints that illuminate the distinct ways that emergency medicine physicians identify with their profession. The research indicates the importance some emergency medicine physicians place on their non-clinical work and societal role, as exemplified in Viewpoint 3; emergency medicine physicians with Viewpoint 1, by contrast, reflect how some emergency medicine physicians may be primarily driven by emphasizing their clinical skill-set; Viewpoint 2 emergency medicine physicians similarly emphasize clinical skill-sets but also highlight the significance of their working environment and departmental support; finally, emergency medicine physicians with Viewpoint 4 affirm that healthy work-life balance should not be overlooked when aiming to understand their professional identities.

Our findings of emergency medicine physicians’ professional identities, particularly amongst those physicians who emphasized Viewpoint 3 (recognition), reinforce a theoretical conceptualisation within identities research whereby identities are multiple and intersecting (Tsouroufli et al. 2011; Monrouxe 2015). Future professional identities research amongst physician populations would benefit from understanding how different professional roles—such as teacher, director, researcher or public health advocate—shape how physician professional identities are conceptualized. Moreover, the value of professional identities that encompass multiple aspects is evident in existing research that has similarly demonstrated that additional non-clinical roles are linked to general practitioners’ (Le Floch et al. 2019) and emergency medicine physicians’ sense of job satisfaction (Clem et al. 2008; Cydulka and Korte 2008).

This finding is especially meaningful when juxtaposed with what is known about emergency medicine physicians who emphasise Viewpoint 2 (coping). Namely, these comparatively young emergency medicine physicians emphasised the stressful elements of their speciality; this is perhaps unsurprising given the abundance of existing literature that highlights the uniquely challenging components of emergency medicine. Daily work challenges are often made worse by insufficient staffing (Camargo et al. 2008), regular overcrowding (Derlet and Richards 2000; Di Somma et al. 2015), sleep disturbances from working night shifts (Machi et al. 2012), and the prevalence of workplace violence (Erdur et al. 2015; Wu et al. 2015; Zafar et al. 2016). The contrast between how emergency medicine physicians with Viewpoint 2 (coping) and Viewpoint 3 (recognition) discuss their professional identities reinforces the value of providing opportunities to develop careers beyond the clinical context. This finding also suggests that developing mentorship opportunities in the emergency department might benefit the development of emergency physicians with relatively less experience. This suggestion takes on added relevance given that our study’s findings on Viewpoint 2 highlights that early career stage emergency medicine physicians emphasize the importance of supportive work environments. The value of mentorship has been demonstrated in research on how to support junior faculty physicians’ in fields wherein the prevalence of work stressors may negatively impact job retention (Weissman 2010) or for physicians who seek to maintain or expand their careers in academia or as physician-leaders (Taylor et al. 2009; Chen et al. 2016).

That said, it would be a disservice to simply interpret the emphasis on stress, expressed by Viewpoint 2 (coping), by contextualizing it with reference to age and lack of experience; such an assessment does not take into account how professional identities are described with reference to the distinct visceral experience of being under pressure and the importance placed on physicality. Thus, a deeper understanding of how *experiential* or *embodied* knowledge (Johnson 1989) underlies emergency medicine physicians’ narratives around their professional identities is critical (Shaw et al. 2018). Therefore, while our study has provided a framework that illuminates the experiences of stress amongst emergency medicine physicians (Viewpoint 2, coping), we believe that future professional identities research, particularly amongst populations who deal with the physical and mental impact of stress on a regular basis, may benefit from engaging in a deeper analysis of their talk within a social constructionist framework (e.g. by attending more closely to the socially and historically-contingent meanings in identities talk) to understand the influences of the wider discourses on how professional identities are constructed (Rees et al. 2019).

Beyond understanding how career trajectories, non-clinical work and negotiating work stress may impact how professional identities are framed, our findings indicate that work-life balance should not be overlooked when exploring professional identities. Particularly given that statements relating to feeling happy outside of work and personal values ranked high amongst all Viewpoints, excluding Viewpoint 1. The importance of a work-life balance demonstrated within our study corroborates existing research that has revealed that an inadequate work-life balance mitigates against the experience of high job satisfaction amongst emergency medicine physicians (Cydulka and Korte 2008) and has become increasingly tied to understanding attrition, burnout and job satisfaction within healthcare (Linzer et al. 2001; Sinsky et al. 2017). Once again, this research implicitly highlights the porous nature of professional and personal spheres as highlighted by intersectionality theoretical perspectives (Tsouroufli et al. 2011; Monrouxe 2015). Thus, within our findings, the intersection of professional and personal identities is abundantly evident in Viewpoint 4 (wellbeing) where participants described their professional identities in relation to identities and fulfilment outside of the professional context. Future organizational initiatives that are intimately aware of the intersections between professional and personal identities (Tsouroufli et al. 2011; Monrouxe 2015) may consider how professional identities may be nurtured by organizational structures that seek to support physicians’ who may prioritize better work-life balance.

That said, enforcing work-life balance is not necessarily appropriate for all physicians. Research identifies that career satisfaction can be attained even when work-life balance is not (Keeton et al. 2007). This is also reflected in our research as emergency medicine physicians with Viewpoint 1 (skills) do not place a strong emphasis on this aspect when describing their professional identities. Further research on physicians’ work-life balance, which is a culturally specific and, at times, a nebulous concept, will be productive to better understanding its significance.

Finally, this research also has educational implications. Existing research indicates that medical students often hold stereotypes about emergency medicine, which may shape their interest, or lack thereof, in pursuing the speciality (Pianosi et al. 2017). Although there is a widely held belief that emergency medicine physicians can attain a “controllable” lifestyle (Hall and Wakeman 1999; Dorsey et al. 2003), it is noteworthy that this perception is not unanimous. For example, some studies reveal that medical students perceive emergency medicine work with poor work-life balance and limited flexibility (Creed et al. 2010; Kawamoto et al. 2016; Pianosi et al. 2017). This perception from students does not necessarily reflect the experiences of practicing emergency medicine physicians, as indicated in our findings, and as has been demonstrated in previous research (Boyd et al. 2009; Pianosi et al. 2017). These findings suggest that there is value in considering what professional identities research might contribute in terms of challenging misconceptions of speciality work culture.

Our research also indicates that emergency medicine physicians’ professional identities are very much affirmed through non-clinical work, such as teaching, research and community outreach, which is a component of an emergency medicine work culture of which students considering the field have relatively poor awareness. It also suggests that there could be benefits to exposing medical students to the types of non-clinical work that emergency medicine physicians participate in, in order for students to develop a more comprehensive grasp on the diverse ways that emergency medicine physicians develop their careers in emergency medicine.

Despite our interesting findings this study has some limitations. Although physicians from five hospitals across different cities in Taiwan were included in this study, all participants were employed by the same hospital group. As mentioned, female emergency medicine physicians over 40 years old are unrepresented. Future studies that incorporate different hospital systems and late career stage female emergency medicine physicians could enhance the discussion on emergency medicine physicians’ notions of professional identities. It is also significant that the study took place towards the end of a collective resignation incident. Physicians participating in the study had inevitably been contemplating issues surrounding emergency medicine physicians’ professional identities and emergency medicine’s work culture, however the specific impact of this event on the four viewpoints is not discernable. Future longitudinal studies that are able to examine the development of professional identities, taking into account the impact of personal and professional crises (Marcia 1966), would yield valuable insight into professional identities.

Notwithstanding these limitations, we believe our study is highly transferable. Although we chose to focus on the professional identities of emergency medicine physicians, our findings offer up an overarching framework that, with alterations, can be used in future studies examining physicians’ professional identities. Moreover our research contributes to the broader field of professional identities research by engaging a subset of physicians who are typically underrepresented in professional identities research: late career stage physicians (Wilson et al. 2013). Furthermore, the use of Q-methodology to tackle identities yielded intriguing findings. Q-methodology interrogates subjectivity rather than focus on primary identities markers, nevertheless our findings are able to reiterate what existing research on identities puts forth (Eckstrand et al. 2016): there is marked value in understanding how alternate elements of identities, in this context specifically age/career stage, impact different viewpoints around professional identities. Finally, these findings are valuable insofar as they provide the necessary building blocks to develop initiatives, build resources and advocate for emergency medicine physicians. At present the research team is drawing on these findings to develop workshops aimed at targeting work stressors and developing resilience in the emergency department.

## Conclusion

By interrogating the dominant perspectives that emergency medicine physicians’ express regarding their professional identities, this research contributes to a fuller understanding of emergency medicine work culture and offers insight into the disparate and shared priorities amongst emergency medicine physicians. Having this awareness does not merely result in greater understanding of emergency medicine physicians, it also provides context regarding how to support physicians more generally, develop interventions that aim to tackle occupational stress, and affect organisational change.
